# Mental healthcare utilization among migrants and Swedish-born adults accounting for probable needs, 2006–2022

**DOI:** 10.1017/S2045796026100717

**Published:** 2026-06-03

**Authors:** Joseph Junior Muwonge, Beata Jablonska, Christina Dalman, Bo Burström, Maria Rosaria Galanti, Anna-Clara Hollander

**Affiliations:** 1 Department of Global Public Health, Karolinska Institute, Stockholm, Sweden; 2 Centre for Epidemiology and Community Medicine, Stockholm Health Care Services, Region Stockholm, Stockholm, Sweden; 3 Transcultural Centre, Stockholm Health Care Services, Region Stockholm, Stockholm, Sweden

**Keywords:** digital services, health equity, immigrants, mental illness, primary care

## Abstract

**Aims:**

Migrants use less mental healthcare than non-migrants, but it is unclear how much this reflects differing needs and whether this gap has changed over time. We examined differences in mental healthcare use by migrant status between 2006 and 2022 while considering probable mental healthcare needs.

**Methods:**

We used data from four cross-sectional surveys conducted in Stockholm County (2006, 2010, 2014 and 2021) in which self-reported need indicators, including psychological distress, were measured. Survey participants, 81,650 adults (18–64), were linked to administrative registries to estimate differences in mental healthcare use (both likelihood and frequency), within 6 months of survey response. Logistic regression and zero-truncated negative binomial regression were used, with survey weights and adjustments for sex, age, income, education, psychological distress (main need-indicator), general health status and long-term limiting illness.

**Results:**

Non-Nordic migrants were more likely to report increased levels of psychological distress but were less likely to use services than non-migrants. The gap in mental healthcare use was initially marginal but increased with adjustment for mental healthcare needs, as well as over time (2006–2022). The odds ratios comparing the likelihood of mental healthcare use between European migrants with Swedish-born individuals decreased from 0.93 (95% confidence intervals: 0.77–1.11) in 2006/2007 to 0.48 (0.39–0.59) in 2021/2022, adjusting for sociodemographic factors and psychological distress. For non-European migrants, the corresponding odds ratios decreased from 0.72 (0.62–0.85) to 0.46 (0.39–0.54). Further adjustments for general health status and long-term limiting illness widened the gap even more. In 2021/2022, the gap was larger in secondary than in primary care and for online than in-office services.

Nordic-born migrants had similar utilization patterns as Swedish-born individuals. Differences in the frequency of outpatient visits between migrants and Swedish-born individuals, conditional on having at least one visit, were marginal. For instance, the rate ratios comparing non-European migrants with Swedish-born individuals changed from 0.67 (0.48–0.93) in 2006/2007 to 0.90 (0.65–1.26) in 2021/2022.

**Conclusions:**

Despite indicating greater needs, non-Nordic migrants faced persistent inequities in mental healthcare access, but differences in intensity/continuity of care were marginal among those who accessed services. Inequities in access grew over the study period and were largest during the COVID-19 pandemic, particularly in access to online mental healthcare services and specialized care. These findings should be interpreted cautiously given potential selection bias from declining survey participation and changes in distress scales.

## Introduction

Mental disorders are common and contribute significantly to the disease burden (WHO, 2020). Beyond their negative impact on individuals and families, these disorders have profound societal effects (Arias *et al.*, 2022). In addition to the general social determinants of mental disorders, such as unemployment and poverty, migrants often face unique challenges in the migration process that may exacerbate their mental disorder risk (Kirkbride *et al.*, 2024). Pre-migration, migrants may face traumatic events like war and conflict, and during migration, they may experience unsafe journeys and poor living conditions (Kirkbride *et al.*, 2024). Post-migration stressors include residence uncertainty, integration difficulties, family separation, social status loss and loneliness (Tinghög *et al.*, 2017; Foverskov *et al.*, 2023; Kirkbride *et al.*, 2024).

Evidence on migrant-related inequalities in the prevalence of mental disorders is mixed (Harris *et al.*, 2019; Kirkbride *et al.*, 2024). Psychotic disorders are more common among migrants (Dykxhoorn *et al.*, 2019), while suicide and substance use disorders are less common in migrants than non-migrants (Harris *et al.*, 2019; Hollander *et al.*, 2020a). Post-traumatic stress disorder (PTSD) is particularly higher among refugees (Fazel *et al.*, 2005; Koenen *et al.*, 2017). Findings on common mental disorders such as depression and anxiety are heterogeneous (Straiton *et al.*, 2024). Although these patterns may be influenced by healthcare-seeking behaviour as studies often rely on diagnostic records.

Healthcare equity is a central policy objective in Sweden and is defined in terms of horizontal equity (equal care for equal needs) and vertical equity (allocating more care to achieve similar outcomes) (Gulliford *et al.*, 2002). The Swedish Health and Medical Services Act mandates that care be provided according to need, prioritizing those with the greatest needs (Sveriges Riksdag, 2017). In practice, this is partly implemented through needs-adjusted capitation reimbursement models, e.g., based on patients’ socioeconomic status and migrant status (Burström *et al.*, 2017).

Despite clear equity goals and minimized financial barriers inherent in publicly financed healthcare systems, migrants use mental healthcare services less than non-migrants (Hollander *et al.*, 2020b). This suggests that services may not be provided equitably (needs-based and regardless of origin), as required by the Swedish Health and Medical Service Act (Sveriges Riksdag, 2017). However, it remains unclear whether the utilization gap reflects lower needs due to the ‘healthy migrant effect’, making access barriers less significant, or whether the gap is larger than previously described given increased needs among migrants. Evidence from the Netherlands (Koopmans *et al.*, 2013) and Northern Ireland (Patel *et al.*, 2021) indicates that migrants underutilize mental healthcare relative to their reported needs, whereas a UK study found that migrants’ lower use of mental healthcare diminishes after adjusting for need indicators (Saunders *et al.*, 2021). Moreover, although related, there is limited evidence on whether migrants experience disparities in gaining access to services or in continuity and intensity of care after entry. Distinguishing between these dimensions (Goff *et al.*, 2025) is important for identifying appropriate intervention strategies.

To our knowledge, it is also unclear how migrant-related inequalities in mental healthcare use have changed over time, particularly in the past 20 years. This period has been marked by major demographic, health system and societal changes, as well as COVID-19-related disruptions, all of which may have influenced service access and equity.

During the past decades, Sweden’s foreign-born population has grown substantially (SCB, 2025a), and its composition has changed notably (SMA, 2023). Finnish-born individuals, once the largest migrant group, have steadily declined in size, while the 2014–2015 refugee influx made Syrian-born individuals the largest group since 2017, followed by Iraqi-born individuals who arrived after earlier conflicts (SCB, 2025b; Swedish Institute, 2026). These changes matter for mental healthcare equity, as migrant groups differ in demographic profiles, language and cultural proximity to Sweden, socioeconomic conditions and mental healthcare needs.

The 2014–2015 refugee influx placed unprecedented strain on the healthcare system, which had to quickly adapt to meet the needs of asylum seekers, including expanding access to interpreters and cultural-competence training (SKR, 2017). Given refugees’ higher risks of severe psychiatric disorders (Kirmayer *et al.*, 2011; Hollander *et al.*, 2016; Tinghög *et al.*, 2017), this surge may also have increased pressure on specialized services.

Sweden has also faced widening economic inequalities (Lancet Reg Health Eur, 2023) and rising poverty rates, particularly among migrants (SCB, 2024). These socioeconomic disadvantages are linked to both higher risks of mental disorders (Kivimäki *et al.*, 2020) and financial barriers to mental healthcare (Molarius *et al.*, 2014) and could lead to further unmet need.

In 2010 (2008 in Stockholm County), Sweden introduced ‘market-oriented’ primary care reforms allowing private provision of tax-funded healthcare and introduced patient choice of providers. Region Stockholm also modified its reimbursement model from mainly capitation to fee-for-service, i.e., tied to production (Burström *et al.*, 2017). Although intended to improve access, concerns were raised that these reforms could worsen equity, e.g., by favouring low-need patients over those with greater needs (who require longer visits) (Burström *et al.*, 2017; Vengberg *et al.*, 2021). While their impact on mental healthcare equity has not been studied, evaluations on general healthcare show mixed results (Beckman and Anell, 2013; Agerholm *et al.*, 2015; MVO, 2015; Burström *et al.*, 2017; Fredriksson and Isaksson, 2022).

Finally, the COVID-19 pandemic disrupted routine service delivery (Duden *et al.*, 2022), limiting opportunities for physical visits and accelerating the expansion of digital services. Use of Region Stockholm’s healthcare app *Alltid öppet* increased from about 40,000 users in early 2020 to over 2 million by 2022 (Region Stockholm, 2022a). Monitoring equity in this context is essential, as lower socioeconomic groups, including migrants, are less likely to use digital services (Wilkens *et al.*, 2024).

Together, these societal and health system shifts, as well as pandemic disruptions, underscore the need to assess how equity in mental healthcare has evolved and to identify groups at risk of persistent unmet needs. Using surveys and registries between 2006 and 2022 in Stockholm County, we describe needs-adjusted mental healthcare use by migrant status. We hypothesized that (1) migrants would use fewer services than individuals born in Sweden, with larger gaps after accounting for estimated needs, and (2) these inequalities would increase over the study period, particularly during COVID-19.

## Methods

### Study setting

Sweden’s decentralized healthcare system is overseen by the 21 counties. We conducted this study in Stockholm County, the most populous county, where 27% of residents are foreign-born compared to 20% nationally (SCB, 2025a). Stockholm residents are free to choose their primary care provider among the publicly financed facilities (public or private). Primary care services treat milder mental health problems (Region Stockholm, 2022b), while secondary care services (where access is mainly referral-based) treat severe mental disorders. For adults, outpatient visits cost a flat rate of 275 Swedish crowns (∼€25/$29) per visit, but fees are waived after about 5 visits within a 12-month period, when the expenditure cap takes effect (Region-Stockholm, 2024). Prescribed medications are subsidized and are subject to a separate expenditure cap (Region-Stockholm, 2024).

### Study design and participants

This observational study followed up participants from four cross-sectional surveys for 6 months in healthcare registries to capture mental healthcare use close to the period of indicating probable mental healthcare needs. The sample includes 110,790 individuals aged 16 and older who participated in one of the *Hälsa Stockholm* surveys in 2006 (response rate 61%), 2010 (55.6%), 2014 (42.3%) and 2021 (48.2%). After excluding adolescents aged 16–17, individuals aged 65 and older and those who had died or emigrated during the 6-month follow-up, the final sample comprised 81,650 individuals. The analytic samples were 27,754 in 2006, 22,463 in 2010, 15,490 in 2014 and 15,943 in 2021.

### Materials/data sources

Data used in this study are from a comprehensive survey-registry linkage database called the Stockholm Public Health Cohort (SPHC), owned by Region Stockholm (Svensson *et al.*, 2012). The SPHC includes both a cohort (followed over time) and cross-sectional samples from the routine *Hälsa Stockholm* surveys conducted between 2002 and 2021. This study used cross-sectional samples from 2006 onwards, as outpatient healthcare registry coverage was incomplete prior to 2006.

The *Hälsa Stockholm* surveys are self-administered public health surveys targeting individuals aged 16 and older living in Stockholm County, identified from the Total Population Registry. Participants are randomly sampled from 38 strata (municipalities or urban districts) to ensure representativeness. The surveys include questionnaires assessing self-rated general health, mental health, lifestyle and health-related behaviours such as alcohol and tobacco use. The questionnaires are available by postal services or online and in translated versions. See Svensson *et al.* (2012) for further details about the SPHC.

Survey data are complemented with registry data. Healthcare records are primarily sourced from Region Stockholm’s healthcare registries, ‘*VAL-databaserna (referred to as VAL)*’, containing visit records from primary (limited coverage before 2014) and secondary outpatient registries, inpatient admissions and data on filled prescriptions. Visit records relate to care received in public and region-financed private facilities in Stockholm County (Svensson *et al.*, 2012). Due to limited data on filled prescriptions before 2016 in VAL, we sourced supplementary data from the National Prescribed Drugs’ Registry. Information on country of birth and socioeconomic status was from the Longitudinal Integration Database for Health Insurance and Labor Market Studies (LISA) and the Total Population Registry.

### Study variables

#### Migrant status

Migrant status was defined based on participants’ country of birth and categorized into four mutually exclusive groups: ‘Sweden’ (born in Sweden), ‘Nordic, other’ (born in other Nordic countries), ‘Europe, other’ (born in other European countries) and ‘Outside Europe’. These categories reflect geographical proximity to Sweden and, while broad, were used to ensure adequate sample sizes for statistical analysis.

#### Mental healthcare use

Mental healthcare utilization was defined as outpatient visits in primary and secondary care or inpatient admissions if a participant (1) had a recorded psychiatric diagnosis, (2) met mental health professionals or (3) filled prescriptions for psychotropic medication based on records from healthcare registries (VAL) and the Prescribed Drugs Registry (*see page 2 of the supplementary material for codes*). This broad definition of mental healthcare use was chosen to ensure that we captured all likely instances of service use related to mental health problems. We analysed both (1) ‘gained/realized’ access to services defined as utilizing services at least once within 6 months of responding to surveys and (2) frequency of outpatient visits during the follow-up period, conditional on at least one visit in primary or secondary outpatient services. We considered both realized access and intensity/continuity of care since the decision to seek mental healthcare and the decision to engage in ongoing care (indicated by the number of visits) may be different.

#### Indicators of mental healthcare needs

Psychological distress, the primary indicator of mental healthcare needs, was measured using the 12-Item General Health Questionnaire (GHQ-12) for the surveys 2006–2014 and the 6-item Kessler Psychological Distress Scale (Kessler-6) used in 2021. The GHQ-12 and Kessler-6 are validated instruments commonly used to screen for non-specific mental health disorders in surveys (Prochaska *et al.*, 2012; Lundin *et al.*, 2017). Both measure how often or how much symptoms have affected a person’s functionality in the past few weeks (GHQ-12) or month (Kessler-6). However, they differ in length, response options and reference period. Additionally, two of the three previous studies comparing the criterion validity of GHQ-12 and Kessler-6 found that Kessler-6 was more accurate in screening for mental health conditions (Furukawa *et al.*, 2003; Patel *et al.*, 2008; Cornelius *et al.*, 2013). The GHQ-12 consists of a score ranging from 0 to 12 (bi-modal scoring 0 0 1 1) while the Kessler-6 has a total score range from 0 to 24 (0 1 2 3 4). Individuals were grouped into three categories based on their scores/severity on the GHQ-12 or Kessler-6: ‘no distress (score of 0 on the GHQ-12 or 0–4 on Kessler-6)’, ‘moderate distress (1–7 on the GHQ-12 or 5–12 on Kessler-6)’ and ‘severe distress (8–12 on the GHQ-12 or 13–24 on Kessler-6)’, following prior Kessler-6 studies (Prochaska *et al.*, 2012) and crosswalks from a study equating the GHQ-12 and Kessler-6 (Lundin *et al.*, 2024).

Self-rated general health status and long-term limiting illness were used as additional indicators of mental healthcare needs, given the presentation of somatic symptoms in patients with mental disorders (Simon *et al.*, 1999) and the established link between physical conditions and mental disorders (Farooqi *et al.*, 2022). Individuals rated their general health status as very good, good, somewhat good, poor or very poor. Long-term limiting illness was measured in two parts: (1) presence of a long-term health problem and (2) whether it impaired daily activities (including work). Those who answered yes to both parts were classified as having a long-term limiting illness, and others (non-missing) were not (*see survey questions on page 2 of the supplementary material*).

#### Covariates

Age, sex (assigned at birth), income and education were collected in the same year as each survey wave and included as covariates to account for group differences and their association with mental healthcare use. Education was divided into seven levels from incomplete primary school to doctoral education. Equivalized disposable household income weighted for household size and composition by Statistics Sweden was categorized into quintiles per wave. Age (continuous) and sex (male/female) were from the Total Population Registry.

### Statistical analysis

Average adjusted predictions from a logistic regression model controlling for age were used to estimate group differences in the prevalence of psychological distress, the primary proxy for mental healthcare needs. A cut-off of ≥3 on the GHQ-12 (2006–2014) or ≥8 on the Kessler-6 (2021) was applied for this purpose (Lundin *et al.*, 2024).

A logistic regression analysis was performed to estimate the association between migrant status and the likelihood of mental healthcare use during follow-up. Both adjusted predicted probabilities of mental healthcare use and odds ratios, with individuals born in Sweden as the reference category, are shown alongside their 95% confidence intervals (CIs).

A zero-truncated negative binomial regression was performed to estimate the association between migrant status and the frequency of visits in outpatient services among those with at least one visit. Both adjusted predicted mean number of visits for each group and rate ratios (RRs) with 95% CIs are shown. Thirteen records of patients who had more than 60 outpatient visits during the 6-month follow-up period were excluded to minimize the effect of outliers on the trend estimates (8/13 were in 2006/2007).

In all analyses, survey (calibrated) weights were used to improve external validity and to estimate more robust standard errors. All analyses were stratified by study period. Model 1 shows crude estimates, while Model 2 is adjusted for age (continuous), sex, household income (in quintiles) and education status (seven levels) to derive estimates that are not biased by group differences in these sociodemographic factors. Model 3 is Model 2 plus adjustment for psychological distress and is reported as the main model, as psychological distress was considered the primary need indicator. In Model 4, additional adjustments were made for self-rated general health status and long-term limiting illness.

In addition, to test for statistical significance of observed changes in estimates across study periods, we performed pooled analyses with interaction terms between migrant status and survey waves. *P*-values from the Wald tests per migrant group are shown.

#### Handling of missing values

Complete case analysis was used, given <1.2% missing data in key variables. However, since about 9.4% was missing for self-rated general health status in 2014, sensitivity analyses were performed: (1) adding a missing category in models and (2) multiple imputations by chained equations (10 datasets).

All analyses were performed in STATA version 18, with graphs produced in R-studio (R version 4.2.2).

## Results

Across survey waves (2006–2021), most participants were born in Sweden (80.1%), but the proportion of non-Nordic migrants increased over time. For instance, migrants from outside Europe increased from 8.9% in 2006 to 14.1% in 2021. In contrast, the proportion of Nordic-born migrants decreased over time (Table 1). Nordic-born migrants were more often female and older adults compared to other groups. Migrant groups generally had lower household income and education status than Swedish-born individuals.

**Table 1.** Characteristics of the study sample in the 2006 survey compared to the 2021 survey Table 1 long description.

	2006				2021			
	Sweden	Nordic, other	Europe	Outside Europe	Sweden	Nordic, other	Europe	Outside Europe
**Total, *N* (%)**	22,521 (81.2%)	1,294 (4.7%)	1,454 (5.2%)	2,479 (8.9%)	12,233 (76.7%)	338 (2.1%)	1,120 (7.0%)	2,251 (14.1%)
**Sex, *N* (%)**								
Males	10,152 (45.1%)	457 (35.3%)	670 (46.1%)	1,190 (48.0%)	5,591 (45.7%)	129 (38.2%)	490 (43.8%)	1,107 (49.2%)
Females	12,369 (54.9%)	837 (64.7%)	784 (53.9%)	1,289 (52.0%)	6,642 (54.3%)	209 (61.8%)	630 (56.3%)	1,144 (50.8%)
**Age, *N* (%)**								
18–29 years	4,334 (19.2%)	65 (5.0%)	246 (16.9%)	507 (20.5%)	2,209 (18.1%)	14 (4.1%)	117 (10.4%)	287 (12.7%)
30–64 years	18,187 (80.8%)	1,229 (95.0%)	1,208 (83.1%)	1,972 (79.5%)	10,024 (81.9%)	324 (95.9%)	1,003 (89.6%)	1,964 (87.3%)
Mean (std)	42.5 (13.2)	49.3 (10.3)	42.9 (12.3)	39.8 (11.3)	43.6 (13.1)	51.3 (11.1)	43.6 (11.1)	43.7 (11.6)
**Education, *N* (%)**								
Primary, ≤9 years	2,711 (12.0%)	243 (18.8%)	275 (18.9%)	462 (18.6%)	893 (7.3%)	23 (6.8%)	93 (8.3%)	299 (13.3%)
Secondary, 10–12 years	9,762 (43.3%)	585 (45.2%)	511 (35.1%)	959 (38.7%)	4,162 (34.0%)	118 (34.9%)	315 (28.1%)	702 (31.2%)
Post-secondary, ≥13 years	10,001 (44.4%)	460 (35.5%)	639 (43.9%)	1,022 (41.2%)	7,139 (58.4%)	185 (54.7%)	662 (59.1%)	1,182 (52.5%)
Missing	47 (0.2%)	6 (0.5%)	29 (2.0%)	36 (1.5%)	39 (0.3%)	12 (3.6%)	50 (4.5%)	68 (3.0%)
**Household income, Mean (SD)**; *’000 Swedish crowns*	246.00 (319.23)	222.34 (194.72)	181.64 (117.63)	154.56 (142.18)	466.43 (725.06)	452.11 (413.13)	363.11 (272.85)	350.26 (1420.80)

### Differences in the prevalence of psychological distress by migrant status

Overall, the age-adjusted prevalence of psychological distress was higher among migrants than Swedish-born individuals across all survey waves (Table 2). In the 2006 wave, 28.7% of the migrants from outside Europe scored ≥3 on the GHQ-12 compared with 18.9% of the Swedish-born individuals (absolute difference = 9.7% [95% CI: 7.6%, 11.8%]). However, group differences were marginal and mainly non-significant in 2014 (e.g., the absolute difference in prevalence between migrants from outside Europe and Swedish-born individuals was 1.5% [−1.1%, 4.2%]). In 2021, when Kessler-6 was used, group differences were larger. For instance, 32.5% of the migrants from outside Europe scored ≥8 on the Kessler-6 scale compared with 24.3% of the Swedish-born individuals (absolute difference = 8.2% [5.9%, 10.6%]; Table 2). Differences between other migrant groups and Swedish-born individuals in 2021 were small (Table 2).

**Table 2.** Age-adjusted prevalence of psychological distress by migrant group and period Table 2 long description.

	2006 Wave (GHQ-12)		2010 Wave (GHQ-12)		2014 Wave (GHQ-12)		2021 Wave (K6)	
Total	%	Absolute differences	%	Absolute differences	%	Absolute differences	%	Absolute differences
Outside Europe	28.7% (26.7–30.7)	9.7% (7.6%, 11.8%)	26.0% (23.9–28.2)	5.3% (3.0%, 7.5%)	27.5% (25.0–30.0)	1.5% (−1.1%, 4.2%)	32.5% (30.3–34.7)	8.2% (5.9%, 10.6%)
Europe	24.8% (22.2–27.4)	5.8% (3.1%, 8.5%)	26.2% (23.4–29.1)	5.5% (2.6%, 8.4%)	22.8% (19.9–25.8)	−3.2% (−6.3%, −0.1%)	27.0% (24.0–30.0)	2.7% (−0.4%, 5.9%)
Nordic, other	19.0% (16.4–21.7)	0.1% (−2.6%, 2.8%)	24.5% (20.9–28.2)	3.8% (0.1%, 7.5%)	26.9% (22.1–31.6)	0.8% (−4.0%, 5.7%)	27.3% (21.0–33.6)	3.0% (−3.4%, 9.4%)
Sweden (ref.)	18.9% (18.3–19.5)	—	20.8% (20.1–21.5)	—	26.0% (25.1–26.9)	—	24.3% (23.4–25.2)	—
**Males**								
Outside Europe	25.2% (22.5–28.0)	10.3% (7.4%, 13.2%)	22.3% (19.3–25.4)	6.0% (2.8%, 9.2%)	25.0% (21.5–28.6)	3.9% (0.1%, 7.7%)	30.0% (26.9–33.1)	9.5% (6.2%, 12.8%)
Europe	20.7% (17.1–24.3)	5.8% (2.1%, 9.5%)	22.3% (18.3–26.3)	6.0% (1.8%, 10.1%)	18.3% (14.3–22.3)	−2.8% (−7.0%, 1.4%)	21.7% (17.5–25.9)	1.2% (−3.2%, 5.6%)
Nordic, other	17.2% (12.8–21.6)	2.3% (−2.1%, 6.8%)	20.9% (15.1–26.7)	4.6% (−1.3%, 10.4%)	23.1% (15.4–30.7)	1.9% (−5.8%, 9.7%)	27.2% (16.8–37.6)	6.7% (−3.8%, 17.1%)
Sweden (ref.)	14.9% (14.1–15.7)	—	16.3% (15.4–17.3)	—	21.2% (19.9–22.5)	—	20.5% (19.3–21.8)	—
**Females**								
Outside Europe	32.3% (29.4–35.2)	9.3% (6.2%, 12.3%)	29.3% (26.3–32.3)	3.8% (0.7%, 7.0%)	30.1% (26.6–33.6)	−0.9% (−4.6%, 2.8%)	35.2% (32.1–38.3)	7.0% (3.7%, 10.4%)
Europe	28.8% (25.1–32.5)	5.7% (1.9%, 9.5%)	29.8% (26.0–33.7)	4.4% (0.4%, 8.4%)	27.7% (23.4–32.1)	−3.2% (−7.8%, 1.3%)	32.0% (27.8–36.2)	3.8% (−0.6%, 8.2%)
Nordic, other	20.2% (16.9–23.6)	−2.9% (−6.3%, 0.6%)	27.0% (22.3–31.7)	1.6% (−3.2%, 6.4%)	29.3% (23.3–35.4)	−1.6% (−7.8%, 4.5%)	27.5% (19.5–35.4)	−0.7% (−8.7%, 7.3%)
Sweden (ref.)	23.1% (22.2–23.9)	—	25.4% (24.4–26.4)	—	31.0% (29.7–32.2)	—	28.2% (26.9–29.4)	—

Probabilities derived using average adjusted predictions from logistic regression adjusting for age.

A cut-off of ≥3 on the GHQ-12 in 2006–2014 or ≥8 on the K6 in 2021 was used for this purpose.

Weighted analysis.

The age-adjusted prevalence of poor rated general health status and long-term limiting illness was higher in migrants than Swedish-born individuals (results not shown).

### Differences in mental healthcare use by migrant status

#### Probability of using mental healthcare services

Table 3 shows the probabilities of using mental healthcare services adjusted for sociodemographic factors and psychological distress. Nordic-born migrants generally had similar probabilities of using mental healthcare services as Swedish-born individuals, and both groups exhibited clear upward trends (2006/2007–2021/2022), whereas migrants from non-Nordic countries consistently had lower (and stable) probabilities of using mental healthcare services than Swedish-born individuals. For instance, in 2006/2007, the probability of using services among migrants from outside Europe was 11.2% compared with 14.5% among Swedish-born individuals (absolute difference = −3.3% [−4.7, −1.8]). In 2021/2022, the probabilities were 12.0% in migrants from outside Europe vs 21.5% in Swedish-born individuals (absolute differences = −9.4% [−11.1, −7.7]). The gap between migrants from other European countries and Swedish-born individuals widened from −0.8% (−2.8, 1.1) in 2006/2007 to −9.1% (−11.2, −6.9) in 2021/2022.

**Table 3.** Predicted probabilities of mental healthcare use by migrant group and period Table 3 long description.

	2006/2007		2010/2011		2014/2015		2021/2022	
Total	%	Absolute differences	%	Absolute differences	%	Absolute differences	%	Absolute differences
Outside Europe	11.2% (9.9–12.5)	−3.3% (−4.7, −1.8)	11.0% (9.6–12.3)	−4.7% (−6.3, −3.2)	11.8% (10.2–13.3)	−6.1% (−7.9, −4.3)	12.0% (10.6–13.5)	−9.4% (−11.1, −7.7)
Europe	13.6% (11.8–15.5)	−0.8% (−2.8, 1.1)	11.3% (9.5–13.0)	−4.5% (−6.3, −2.6)	12.6% (10.4–14.7)	−5.3% (−7.6, −2.9)	12.4% (10.4–14.4)	−9.1% (−11.2, −6.9)
Nordic, other	13.4% (11.4–15.4)	−1.1% (−3.1, 1.0)	13.8% (11.6–16.1)	−1.9% (−4.2, 0.4)	17.0% (13.6–20.3)	−0.9% (−4.3, 2.5)	22.5% (17.6–27.4)	1.0% (−3.9, 6.0)
Sweden (ref.)	14.5% (13.9–15.0)	—	15.7% (15.1–16.3)	—	17.8% (17.1–18.6)	—	21.5% (20.6–22.3)	—
**Males**								
Outside Europe	7.9% (6.3–9.4)	−3.3% (−5.1%, −1.5%)	9.2% (7.3–11.1)	−2.2% (−4.3%, −0.1%)	8.2% (6.3–10.1)	−4.9% (−7.2%, −2.7%)	8.3% (6.5–10.1)	−7.4% (−9.5%, −5.2%)
Europe	10.7% (8.3–13.1)	−0.5% (−3.0%, 2.1%)	8.4% (6.2–10.6)	−3.0% (−5.3%, −0.6%)	8.2% (5.4–11.0)	−4.9% (−7.9%, −1.8%)	7.7% (5.1–10.2)	−8.0% (−10.8%, −5.2%)
Nordic, other	9.9% (6.8–12.9)	−1.3% (−4.5%, 1.8%)	7.1% (4.2–10.0)	−4.3% (−7.3%, −1.2%)	12.1% (7.2–16.9)	−1.1% (−6.0%, 3.8%)	18.2% (10.3–26.1)	2.6% (−5.4%, 10.5%)
Sweden (ref.)	11.2% (10.4–11.9)	—	11.4% (10.5–12.2)	—	13.1% (12.0–14.2)	—	15.7% (14.5–16.8)	—
**Females**								
Outside Europe	14.5% (12.4–16.6)	−3.3% (−5.6%, −1.1%)	12.9% (10.9–14.8)	−7.2% (−9.4%, −5.0%)	15.4% (12.9–17.9)	−7.2% (−10.0%, −4.4%)	15.9% (13.5–18.2)	−11.4% (−14.1%, −8.7%)
Europe	16.4% (13.6–19.2)	−1.4% (−4.3%, 1.5%)	14.3% (11.6–16.9)	−5.8% (−8.6%, −3.0%)	17.0% (13.7–20.3)	−5.6% (−9.2%, −2.1%)	17.0% (14.0–20.1)	−10.3% (−13.6%, −6.9%)
Nordic, other	16.9% (14.1–19.8)	−0.9% (−3.8%, 2.0%)	19.2% (15.9–22.6)	−0.8% (−4.3%, 2.7%)	21.8% (17.0–26.5)	−0.9% (−5.7%, 4.0%)	27.5% (20.9–34.1)	0.2% (−6.5%, 6.9%)
Sweden (ref.)	17.8% (17.0–18.6)	—	20.0% (19.1–21.0)	—	22.6% (21.5–23.8)	—	27.3% (26.1–28.5)	—

Probabilities derived using average adjusted predictions from logistic regression adjusting for age, income (quintiles), education (seven levels), psychological distress (three levels) and sex (in unstratified analyses).

Weighted analysis.

Figures S1–S2 and Table S1 (supplementary material) show trends in mental healthcare use by healthcare level and contact type. Visits to secondary outpatient care, collection of psychotropic medication and in-office contacts followed similar trends as those observed above: probabilities were either stable or declined among migrants from non-Nordic countries in 2021/2022 compared to 2014/2015 but increased among Nordic-born migrants and Swedish-born individuals. In contrast, primary care use and online contacts increased for all groups but at a higher rate for Nordic-born migrants and Swedish-born individuals.

#### Relative differences in mental healthcare use by migrant status

**Figure 1.**
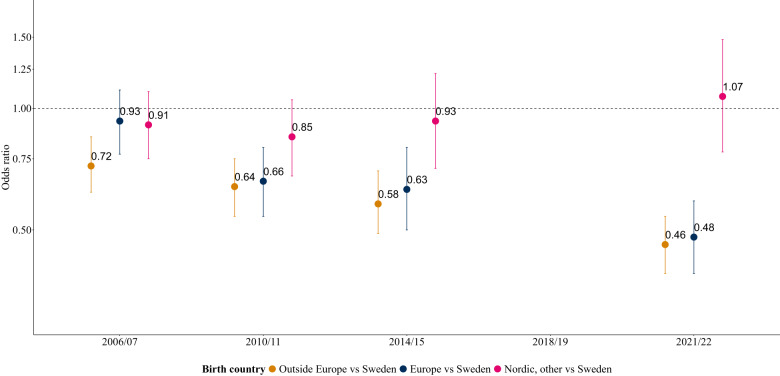
Odds ratios comparing mental healthcare use between migrant groups and Swedish-born individuals across survey waves, from weighted analyses adjusted for sociodemographic variables and psychological distress. See Table S2 for crude and stepwise adjustments for sociodemographic and need indicators. Figure 1 long description.

In 2006/2007, only migrants from outside Europe were less likely to use services than Swedish-born individuals, adjusting for sociodemographic factors (see Table S2). Adjusting for psychological distress led to larger differences between all groups and Swedish-born individuals, but only migrants from outside Europe were significantly less likely to utilize mental healthcare services (Fig. 1). In subsequent waves, both migrant groups from (non-Nordic) Europe and from outside Europe were significantly less likely to utilize services compared with Swedish-born individuals. In addition, these differences significantly increased over time (Wald test, *p*-value < 0.0001), such that by 2021/2022, both groups utilized services at about half the rate of individuals born in Sweden (odds ratios = 0.46 [0.39; 0.54] outside Europe vs Sweden and 0.48 [0.39; 0.59] Europe vs Sweden). Conversely, there were no significant differences in mental healthcare use between Nordic-born migrants and Swedish-born individuals when adjusting for sociodemographic factors and psychological distress (Wald test, *p*-value = 0.9632). In addition, analyses adjusting for additional need-indicators depicted similar trends – with a widening gap between non-Nordic migrant groups and Swedish-born individuals (Table S2).

Sex- and age-stratified analyses generally depicted comparable trends to those observed above (Table S3).

**Figure 2.**
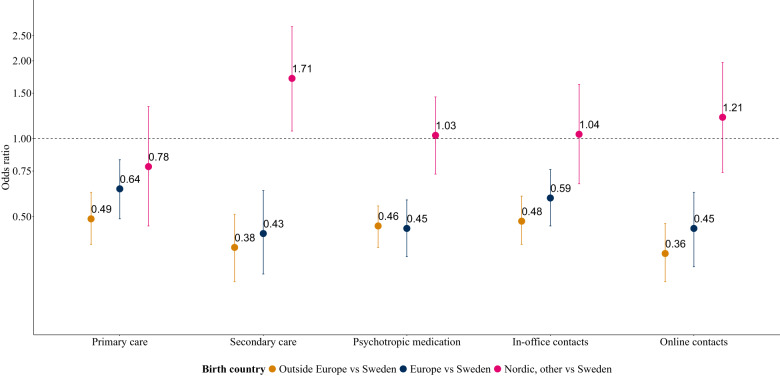
Odds ratios comparing mental healthcare use between migrant groups and Swedish-born individuals by healthcare level and type of contact in 2021/2022, from weighted analyses adjusted for sociodemographic variables and psychological distress. Figure 2 long description.

Figure 2 shows adjusted odds ratios of mental healthcare use by healthcare level and type of contact for 2021/2022. Although findings were comparable by healthcare level and type of contact, differences by migrant status were larger in secondary (outpatient) care than in primary care and for online contacts than in-office contacts (Table S4).

### Differences in the number of outpatient visits, conditional on having at least one visit

**Figure 3.**
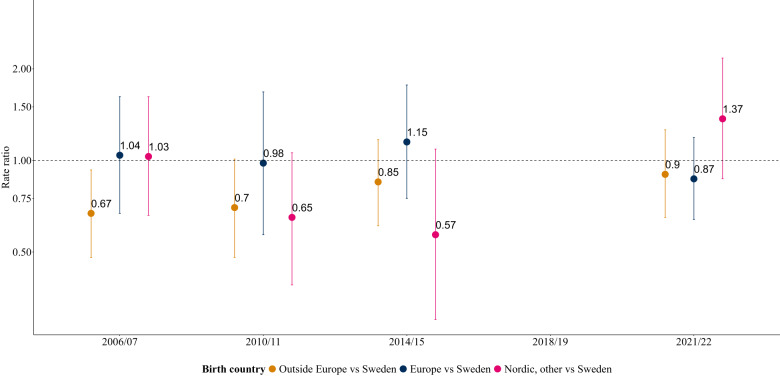
Rate ratios comparing the frequency of outpatient visits between migrant groups and Swedish-born individuals across survey waves, conditional on having at least one visit. Analyses are weighted and adjusted for sociodemographic variables and psychological distress. Figure 3 long description.

Between 2006/2007 and 2014/2015, there was a decline in the predicted number of outpatient visits among mental healthcare users in each group, followed by an increase in 2021/2022 (except for European migrants; Table S5). Differences between groups at each period were marginal, but migrants from outside Europe had slightly fewer visits than Swedish-born individuals (Table S5). Figure 3 shows the RRs of the frequency of outpatient visits comparing migrant groups and Swedish-born individuals, conditional on having at least one visit. Initially (2006/2007), migrants from outside Europe visited outpatient services less frequently than Swedish-born individuals (RR = 0.67 [0.48; 0.93]), but in subsequent waves, these differences were attenuated and not significant (e.g., in 2021/2022: RR = 0.90 [0.65; 1.26]). However, period-specific estimates were not significantly different (Wald test, *p*-value = 0.7139). Analyses adjusting for additional need indicators depicted similar trends (Table S6). Due to few cases per study period, age- and sex-stratified analyses were not performed.

### Results from sensitivity analysis and post hoc analysis

Because access to secondary outpatient care is mainly referral-driven, a post hoc analysis restricted to individuals in contact with primary care was performed and showed similar but slightly attenuated estimates (see notes in Table S4).

Using missing values as categories in the regression analysis and multiple imputations returned similar estimates (Table S7).

We checked the level of psychological distress among individuals in contact with mental healthcare services and found that migrants had higher distress levels than Swedish-born mental healthcare users. For instance, in 2021/2022, among individuals in contact with services, migrants from outside Europe had a mean Kessler-6 score of 9.86 (SD: 6.35) compared to 7.76 (SD: 5.31) among Swedish-born individuals (Table S8).

Additional analyses were performed to assess the robustness of the observed trends given the change in the distress scale from GHQ-12 in 2014 to Kessler-6 in 2021 (see Table S9, A–F). Models included adjustments using only long-term limiting illness and general health status as need indicators (consistently measured across waves), analyses using raw distress scales (before equating), binary cut-offs and the sample distribution (deciles and ranks instead of cut-offs). All models showed a similar widening of inequalities between 2014/2015 and 2021/2022.

## Discussion

This study aimed to examine migrant-related inequalities in mental healthcare use among adults while accounting for their probable mental healthcare needs. Non-Nordic migrants indicated higher mental healthcare needs but used less mental healthcare than Swedish-born individuals. These differences increased over the study period and were largest during the COVID-19 pandemic. However, conditional on gained access in outpatient services, there were mostly no significant inequalities in the frequency of visits.

Consistent with previous studies from our group, non-Nordic migrants had poorer ‘gained’ access to mental healthcare services than Swedish-born individuals (Hollander *et al.*, 2020b). In this study, in which we for the first time have indicators of likely mental healthcare need, we found that migrants indicated more needs than Swedish-born individuals, providing no evidence of a ‘healthy migrant effect’. Accordingly, adjusting for probable mental healthcare needs revealed even greater migrant inequalities in mental healthcare use, suggesting that the access gap is wider than previously described. This pattern aligns with observed gaps in outpatient service use in the Netherlands (Koopmans *et al.*, 2013) and psychotropic medication use in Northern Ireland (Patel *et al.*, 2021) among migrant groups with likely mental healthcare needs.

Why migrants with probable needs are less likely to access mental healthcare services than non-migrants was not investigated in this study but may be explained by the Levesque framework (Levesque *et al.*, 2013). Inequities may reflect differences in perceived need and in ability to seek, reach, pay for and engage with services (Levesque *et al.*, 2013). For instance, self-stigma, low mental health literacy and beliefs about mental illness or service eligibility may limit perceived need and willingness to seek help (Dixon-Woods *et al.*, 2006). In our study, migrants who accessed services had reported more severe distress than Swedish-born individuals, which may indicate a higher threshold for seeking care or delayed access. Limited service awareness may also reduce the ability to reach care (Dixon-Woods *et al.*, 2006). Additionally, structural factors such as logistical, financial and communication barriers and low transcultural awareness in the healthcare system may restrict migrants’ ability to reach, pay for and engage with services (Dixon-Woods *et al.*, 2006; Levesque *et al.*, 2013).

Although non-Nordic European migrants had similarly lower odds of accessing services as non-European migrants in this study, the underlying mechanisms may differ but could not be examined in the present study. For instance, non-Nordic European migrants may be more likely to seek mental healthcare in their home countries due to geographical proximity and greater familiarity with the healthcare system, which may reduce their engagement with services in Sweden.

Interestingly, the disparities in intensity or continuity of care were not obvious among survey participants with at least one outpatient visit; in fact, differences in the number of outpatient visits were mostly marginal and non-significant. This contrasts with Nordic studies reporting fewer visits among migrants than non-migrants (Abebe *et al.*, 2017; Kieseppä *et al.*, 2020). One explanation is that those studies examined total populations, whereas our study, which used survey participants where recent migrants are underrepresented, may underestimate true inequalities. In addition, those studies relied only on secondary care records, while we also included primary care, where inequities were less pronounced (based on data from 2021/2022). However, this finding is important in two ways. Firstly, it suggests that once migrants access services, they continue to use services and at a relatively similar rate as Swedish-born individuals, assuming the quality or content of a visit is the same for migrants as for Swedish-born individuals. Secondly, it suggests that addressing migrants’ lower use of services may be most effective by reducing barriers to seeking and accessing care. A total population study (Hollander *et al.*, 2020b), which accounted for time living in Sweden, found that most disparities diminish over time, likely as migrants become more familiar with the healthcare system.

Inequities faced by migrants from non-Nordic countries were smaller in primary care than in secondary care, probably due to primary care’s community presence and lower stigma associated with such services. However, migrants’ lower access to primary care may partially explain the larger differences in secondary care since access is mainly referral-based. A post hoc analysis among individuals with access to primary care, although based on a smaller sample size, showed similar but slightly attenuated migrant inequities in secondary care. Alternatively, inequities in referral patterns from primary care (such as migrants being less likely referred to specialists than the majority native-born population) might explain the larger inequities in secondary care (Dixon-Woods *et al.*, 2006; Duveau *et al.*, 2023).

This study found that inequalities in access widened in 2021/2022 compared with earlier periods, with similar patterns across age and sex groups. The widening gaps may partly be due to COVID-19-related system disruptions, particularly the rapid digitalization of healthcare services. Although use of digital mental healthcare services increased for all groups in 2021/2022 compared with 2014/2015, the increase was substantially greater among non-migrants. Notably, access gaps in 2021/2022 were larger for digital services than physical services. This is concerning given that digital services were meant to enhance access, particularly during COVID-19 when physical visits were restricted.

Poorer access to digital services among migrants is consistent with previous research, as a pre-pandemic study in Sweden revealed that lower socioeconomic groups, including migrants, underutilized digital services (Wilkens *et al.*, 2024). Likely barriers include limited access to user-friendly devices, complicated log-in procedures (e.g., BankID access and triage questions), low digital literacy, language barriers when services are available only in Swedish, privacy concerns (including limited private space for video sessions), limited awareness of available digital options and a preference for in-person care (Bol *et al.*, 2018; Muli *et al.*, 2025).

Therefore, the observed trend aligns with the ‘Inverse Equity Hypothesis’, whereby more affluent groups adopt new health interventions/technologies earlier than other groups, initially widening inequities. As digital technologies increasingly become integrated into traditional mental healthcare services, this finding underscores the need for a more inclusive digitalization of mental healthcare (Kalman *et al.*, 2023).

## Strengths

By linking surveys with registry data, we were able to account for indicators of mental healthcare needs, addressing a key limitation of previous studies that only used registry data. To the best of our knowledge, this is the first study in Sweden to assess migrant inequalities in mental healthcare use based on a comprehensive healthcare database including primary care records, where most adults receive mental healthcare services. The large sample size allowed us to disentangle differences in inequities by healthcare level, and type of visit, thereby identifying areas where migrants might be at most disadvantage. Using data from four periods, including during COVID-19, we were able to analyse temporal changes in migrant inequities.

## Limitations

There are several limitations to consider while interpreting our results. First, there is a lack of data from 2018/2019 (pre-pandemic), which limits our assessment of whether the changes observed in 2021/2022 were related to pandemic disruptions. Second, the lower coverage of primary care use in the earlier study period may bias our trend estimates due to potential outcome misclassification in the earlier waves since mental healthcare is mainly provided in primary care (Sundquist *et al.*, 2017; Schmidt-Mende *et al.*, 2025). Nevertheless, this will likely not impact our estimates since we expect non-differential misclassification (i.e., ‘underdiagnosis’ in all groups), and because primary care access was likely captured through psychotropic medication records, as about 65% of antidepressant prescriptions are issued in primary care (Swedish National Board of Health and Welfare) (Socialstyrelsen, 2021).

Third, selection bias due to systematic non-response may bias our equity assessments since migrants are less likely to respond. For instance, if migrant participants are more likely to use services than non-participants, our observed inequalities would be an underestimate of the true inequalities. This is the likely direction of bias, as shown in a study by Agerholm *et al.* (2016), which compared migrant inequalities in healthcare utilization among survey participants with inequalities in the total population and found that inequalities were attenuated among survey participants. Moreover, the falling response rates over time, a common issue in surveys, may further bias trend estimates.

Fourth, the change from GHQ-12 (used in 2006–2014) to Kessler-6 (2021) may have influenced inequity trends, as Kessler-6 better captures needs (Furukawa *et al.*, 2003; Patel *et al.*, 2008; Cornelius *et al.*, 2013). However, this impact is likely minimal because crude estimates and those adjusted only for sociodemographic factors showed similar trends. In addition, sensitivity analyses using the consistently collected need variables, long-term limiting illness and general health status, also showed a widening of inequalities between 2014/2015 and 2021/2022. Additional analyses including using the raw distress scales (before equating) and using binary cut-offs (≥3 on the GHQ-12 or ≥8 on the Kessler-6 and ≥8 on the GHQ-12 or ≥13 on the Kessler-6 based on crosswalks) similarly indicated a widening of inequalities between 2014/2015 and 2021/2022.

In addition, potential misclassification due to group differences in the expression and reporting of distress symptoms could bias need-adjusted inequalities; for example, groups that underreport or overreport distress may appear to over- or underutilize mental healthcare services. Moreover, if the meaning of distress changes over time, this may bias the interpretation of the temporal trends.

These findings confirm that non-Nordic migrants use mental healthcare less than others with comparable needs. The disparities are obvious in terms of gained access but less apparent in the frequency of use, conditional on gained access. Therefore, interventions to improve migrants’ access to services should be prioritized, such as community-based programmes for newly arrived migrants and transcultural training for providers (Svanholm *et al.*, 2020; Place *et al.*, 2021; Bäärnhielm and Schouler-Ocak, 2022).

## Conclusion

This survey-registry linked study found that, despite greater needs, non-Nordic migrants faced persistent inequities in mental healthcare access, but differences in continuity of care were marginal among those who accessed services. Inequities in access widened over the study period and were largest during the COVID-19 pandemic, particularly for online services and specialized care. Although interpretation should be cautious due to potential selection bias and change in distress scales, the findings reinforce the need for targeted interventions to improve migrant access and reduce persistent inequities.

## Supporting information

10.1017/S2045796026100717.sm001 Muwonge et al. supplementary material Muwonge et al. supplementary material

## Data Availability

Request SPHC data: Hälsa Stockholm - för forskare (regionstockholm.se).

## Supplementary material

The supplementary material for this article can be found at https://doi.org/10.1017/S2045796026100717.

## Table 1 Long description

The table compares survey participant characteristics in 2006 and 2021 by region of birth: Sweden, other Nordic countries, other European countries, and outside Europe. In 2006, most participants were born in Sweden (22,521; 81.2%), with 8.9% born outside Europe; in 2021, Sweden-born decreased to 12,233 (76.7%) and outside-Europe increased to 2,251 (14.1%). Sex distributions were similar across years, with women slightly more common in most groups; for example, Sweden-born were 54.9% female in 2006 and 54.3% female in 2021. The age profile shifted older for those born in other Nordic countries, with mean age rising from 49.3 to 51.3 years, while the share aged 18 to 29 remained low in that group (about 5% in 2006 and about 4% in 2021). Post-secondary education became more common in 2021, rising among Sweden-born from 44.4% to 58.4% and also increasing in the other groups (for example, Europe from 43.9% to 59.1%). Primary education decreased in most groups, notably among Sweden-born from 12.0% to 7.3%. Mean household income (in thousands of Swedish crowns) was higher in 2021 than 2006 for all birth regions, though variability was large, especially for those born outside Europe in 2021. Percentages are within each birth-region column for each year, and differences may reflect changes in sample size and composition between surveys.

Navigate back to Table 1.

## Table 2 Long description

The table reports age-adjusted prevalence of psychological distress for four origin groups (outside Europe, Europe, other Nordic, and Sweden as the reference) across survey waves in 2006, 2010, 2014, and 2021, with results also split by males and females. For each wave, it lists the percent distressed with confidence intervals and the absolute difference compared with Sweden. In the total population, migrants from outside Europe show consistently higher distress than Sweden in every wave, rising from about 29% in 2006 to about 33% in 2021, with gaps versus Sweden ranging from about 1.5 to 9.7 percentage points. European migrants also have generally higher prevalence than Sweden-born people in 2006, 2010, and 2021, but in 2014 they have slightly lower prevalence than Sweden-born people (about 23% versus 26%). Other Nordic migrants are close to Sweden in 2006, then higher in later waves, reaching about 27% in 2014 and 2021. By sex, females have higher prevalence than males within each origin group in all waves; in 2021, females from outside Europe are about 35% versus about 30% for males from outside Europe, while Sweden is about 28% for females and about 21% for males. Comparisons across waves should be interpreted cautiously because the 2021 measure uses a different distress scale and cutoff than earlier waves, and estimates are model-based and weighted.

Navigate back to Table 2.

## Table 3 Long description

The table reports predicted probabilities, with confidence intervals, of using mental healthcare across four time periods for migrants from outside Europe, Europe, other Nordic countries, and Sweden as the reference group, shown for the total population and separately for males and females. In the total population, Sweden increases from 14.5 percent in 2006/2007 to 21.5 percent in 2021/2022, while other Nordic migrants rise from 13.4 to 22.5 percent. Over the same span, migrants from outside Europe remain around 11 to 12 percent and migrants from Europe around 11 to 14 percent, both consistently below Sweden. The “absolute differences” columns indicate gaps versus Sweden that generally widen over time for Europe and outside-Europe groups, reaching about 9 percentage points lower than Sweden by 2021/2022. For males, Sweden rises from 11.2 to 15.7 percent; other Nordic males increase to 18.2 percent by 2021/2022, while Europe and outside-Europe males stay near 8 percent and become further below Sweden over time. For females, levels are higher than for males in every group; Sweden rises from 17.8 to 27.3 percent and other Nordic females to 27.5 percent, while Europe and outside-Europe females remain lower at about 17.0 and 15.9 percent in 2021/2022. Estimates come from adjusted, weighted logistic regression predictions, so differences reflect modeled probabilities after accounting for demographic and socioeconomic factors rather than raw rates.

Navigate back to Table 3.

## Figure 1 Long description

Odds ratio A dot and error bar chart with five x-axis categories: 2006 slash 07, 2010 slash 11, 2014 slash 15, 2018 slash 19, 2021 slash 22. The y-axis is labeled Odds ratio, with tick labels 0.50, 0.75, 1.00, 1.25, 1.50. A horizontal dashed reference line is drawn at 1.00. Legend text at the bottom: Birth country; Outside Europe vs Sweden; Europe vs Sweden; Nordic, other vs Sweden. At 2006 slash 07, three points are labeled 0.72 (Outside Europe vs Sweden), 0.93 (Europe vs Sweden) and 0.91 (Nordic, other vs Sweden). Each point has a vertical error bar. At 2010 slash 11, three points are labeled 0.64 (Outside Europe vs Sweden), 0.66 (Europe vs Sweden) and 0.85 (Nordic, other vs Sweden). Each point has a vertical error bar. At 2014 slash 15, three points are labeled 0.58 (Outside Europe vs Sweden), 0.63 (Europe vs Sweden) and 0.93 (Nordic, other vs Sweden). Each point has a vertical error bar. At 2018 slash 19, no plotted points or value labels are shown. At 2021 slash 22, two points are labeled 0.46 (Outside Europe vs Sweden) and 0.48 (Europe vs Sweden), each with a vertical error bar. A third point is labeled 1.07 (Nordic, other vs Sweden) with a vertical error bar.

Navigate back to Figure 1.

## Figure 2 Long description

The y-axis is labeled Odds ratio, with tick labels 0.50, 0.75, 1.00, 1.50, 2.00 and 2.50. A dashed horizontal reference line is drawn at 1.00. The x-axis shows five categories: Primary care; Secondary care; Psychotropic medication; In-office contacts; Online contacts. A legend labeled Birth country shows three series: Outside Europe vs Sweden; Europe vs Sweden; Nordic, other vs Sweden. Primary care: Outside Europe vs Sweden is labeled 0.49. Europe vs Sweden is labeled 0.64. Nordic, other vs Sweden is labeled 0.78. Secondary care: Outside Europe vs Sweden is labeled 0.38. Europe vs Sweden is labeled 0.43. Nordic, other vs Sweden is labeled 1.71. Psychotropic medication: Outside Europe vs Sweden is labeled 0.46. Europe vs Sweden is labeled 0.45. Nordic, other vs Sweden is labeled 1.03. In-office contacts: Outside Europe vs Sweden is labeled 0.48. Europe vs Sweden is labeled 0.59. Nordic, other vs Sweden is labeled 1.04. Online contacts: Outside Europe vs Sweden is labeled 0.36. Europe vs Sweden is labeled 0.45. Nordic, other vs Sweden is labeled 1.21. Each plotted point has a vertical error bar.

Navigate back to Figure 2.

## Figure 3 Long description

Rate ratios comparing the frequency of outpatient visits between migrant groups and Swedish-born individuals across survey waves, conditional on having at least one visit. A dot and error bar chart with a dashed horizontal reference line at 1.00. The x-axis is labeled 2006/07, 2010/11, 2014/15, 2018/19, 2021/22. The y-axis is labeled Rate ratio, with labeled ticks at 0.50, 0.75, 1.00, 1.50 and 2.00. A legend reads: Birth country; Outside Europe vs Sweden; Europe vs Sweden; Nordic, other vs Sweden. At 2006/07, three points are labeled 0.67 (Outside Europe vs Sweden), 1.04 (Europe vs Sweden) and 1.03 (Nordic, other vs Sweden), each with a vertical error bar. At 2010/11, two points are labeled 0.7 (Outside Europe vs Sweden) and 0.98 (Europe vs Sweden), each with a vertical error bar. A third point is labeled 0.65 (Nordic, other vs Sweden) with a vertical error bar. At 2014/15, three points are labeled 0.85 (Outside Europe vs Sweden), 1.15 (Europe vs Sweden) and 0.57 (Nordic, other vs Sweden), each with a vertical error bar. At 2018/19, no plotted points are shown. At 2021/22, three points are labeled 0.9 (Outside Europe vs Sweden), 0.87 (Europe vs Sweden) and 1.37 (Nordic, other vs Sweden), each with a vertical error bar.

Navigate back to Figure 3.
