# Supplementary material for: Mental healthcare utilization among migrants and Swedish-born adults accounting for probable needs, 2006–2022
Source: Epidemiol Psychiatr Sci. 2026 Jun 3;35:e33. doi: 10.1017/S2045796026100717 (PMC13247915; doi:10.1017/S2045796026100717)
Supplement: Muwonge et al. supplementary material [file S2045796026100717sup001.docx]

**Supplementary material for manuscript titled: Mental healthcare utilization among migrants and Swedish-born adults accounting for probable needs, 2006-2022**

J.J. Muwonge^1,2^ (ORCID: 0000-0002-9219-9752), B. Jablonska^1,2^ (ORCID: 0000-0002-0246-6643), C. Dalman^1,2^ (ORCID: 0000-0002-3579-2357), B. Burström^1,2^ (ORCID: [0000-0001-5770-9422](https://orcid.org/0000-0001-5770-9422)), M.R. Galanti^1^ (ORCID: 0000-0002-7805-280X), A-C. Hollander^1,3^ (ORCID:[0000-0002-1246-5804](https://orcid.org/0000-0002-1246-5804))

1. Department of Global Public Health, Karolinska Institute, Stockholm, Sweden, 171 77 Stockholm
2. Centre for Epidemiology and Community Medicine, Stockholm Health Care Services, Region Stockholm, Stockholm, Sweden, Solnavägen 1e, P.O Box 45436, 104 31 Stockholm.
3. Transcultural Centre, Stockholm Health Care Services, Stockholm, Region Stockholm, Stockholm, Sweden, Solnavägen 1e, 113 65 Stockholm.

Corresponding author

Name: Joseph Junior Muwonge

Tel: +46761534803

Contact: [joseph.junior.muwonge@ki.se](mailto:joseph.junior.muwonge@ki.se)

**Table of contents**

[**Methods’ supplements** 2](#_Toc225778257)

[*Measurement of Mental Healthcare use* 2](#_Toc225778258)

[*Additional need indicators* 2](#_Toc225778259)

[*Adjusting for both income and education* 2](#_Toc225778260)

[*Handling missing values* 2](#_Toc225778261)

[*Survey (calibrated) weights* 2](#_Toc225778262)

[**Results** 3](#_Toc225778263)

[*Predicted probabilities in mental healthcare utilization by level and type of contact (2006-2022)* 3](#_Toc225778264)

[*Odds ratios comparing mental healthcare utilization between migrant groups and Swedish-born individuals (2006-2022)* 5](#_Toc225778265)

[*Sex- and age-stratified analyses of differences in mental healthcare utilization between migrant groups and Swedish-born individuals (2006-2022)* 6](#_Toc225778266)

[*Odds ratios comparing mental healthcare utilization between migrant groups and Swedish-born individuals by healthcare level and type of contact (2021/2022)* 7](#_Toc225778267)

[*Predicted number of outpatient visits by group, conditional on at least one visit (2006-2022)* 7](#_Toc225778268)

[*Rate ratios comparing number of outpatient visits between migrant groups and Swedish-born individuals (2006-2022)* 8](#_Toc225778269)

[*Sensitivity analysis comparing results based on complete case analysis and methods for handling missing data* 9](#_Toc225778270)

[*Mean of psychological distress among mental healthcare users by migrant status (2006-2022)* 9](#_Toc225778271)

[*Robustness checks due to change of instrument from GHQ-12 to Kessler 6* 10](#_Toc225778272)

# **Methods’ supplements**

## *Measurement of Mental Healthcare use*

- Psychiatric diagnoses were based on the tenth revision of the International Classification of Diseases (ICD-10) diagnostic codes: F00-F99, X60-X84, Z72820, Z915, G47, R45851, and T1491.
- Psychotropic medication was identified using the following Anatomical Therapeutic Chemical code (ATC) codes: N05A, N05B, N05C, N06A, N06B, N07BB, and N07BC
- Psychosocial support in primary and secondary outpatient care was identified using the following codes: Uppdragtyp (301, 320), KLIN (950, 951, 955), and VDG1-5 (74, 75, 96; meeting with a psychologist, counsellor, and psychotherapist).
- Healthcare level (Vårdnivå: vardniva) was identified using the codes: ‘01’ for primary care and ‘02’ for secondary care.
- Visit type (besökstyp: btyp) was identified using the following codes: btyp in ('6', '9', 'U', 'W', 'Y') for digital visits and btyp in ('0','1','2','8','A', 'B', 'D', 'E', 'F', 'G', 'H', 'K', 'L', 'M', 'N', 'P') for physical visits.

## *Additional need indicators*

General health status was measured by the question, *“How do you rate your general health status?”* with responses 1*) very good, 2) good, 3) somewhat good, 4) poor, and 5) very poor*. Long-Term Limiting illness was measured using two questions: (a) *“Do you have any long-term sickness, discomfort following an accident, reduced physical function, or any other long-term health problem?”* (with responses *1 = yes or 2 = no)* and (b) if yes, have these problems resulted in your ability to work being impaired or hindered you from other daily activities? (with responses *1 = yes, to a large extent; 2 = yes, to some extent; 3 = no*). Respondents answering "yes" to both questions were coded as having a limiting illness; all others (non-missing) were coded as not having a limiting illness.

## *Adjusting for both income and education*

Since education and income may be highly correlated and therefore lead to multicollinearity, we tested for multicollinearity using Variance Inflation Factors (VIFs; for each period, the VIF was approximately 1,04) and estimates were similar when adjusting only for income and when including education to the model, we therefore decided to adjust for both income and education to avoid “omitted variable bias”.

## *Handling missing values*

Complete case analysis was performed due to a low overall proportion of missing in the main variables (<1.2%). However, since the proportion of item missing in self-rated general health status was high in the 2014 wave (~ 9.4% in 2014 compared to 1,0 % in 2006, 0.9% in 2010, 0.3% in 2021) and was missing at random (*MAR; education, income, distress, and long-term illness were significantly associated with missing on self-rated general health status in 2014*), we performed sensitivity analysis to check for the robustness of the Model 3 analysis (adjusting for self-rated general health status). 1) We added missing as a dummy variable in the regression models; 2) we used multiple imputation by chained equations to create ten imputed datasets, with all fully observed variables included in the regression analysis added in the imputation model.

## *Survey (calibrated) weights*

Because survey weights had been calibrated using among other variables, information about country of birth (our independent variable), we performed sensitivity analysis to check whether this affected our results. The results were generally similar, for instance odds ratios comparing mental healthcare use in non-European migrants to Swedish-born individuals was OR = 0∙73 (95% CI: 0∙64; 0∙84) in 2006/2007 without weights, and OR = 0∙72 (0∙62; 0∙85) with weights. In 2021/2022, the odds ratios were OR = 0∙44 (0∙38; 0∙50) without weights and 0∙46 (0∙39; 0∙54) with weights.

# **Results**

## *Predicted probabilities in mental healthcare utilization by level and type of contact (2006-2022)*

**
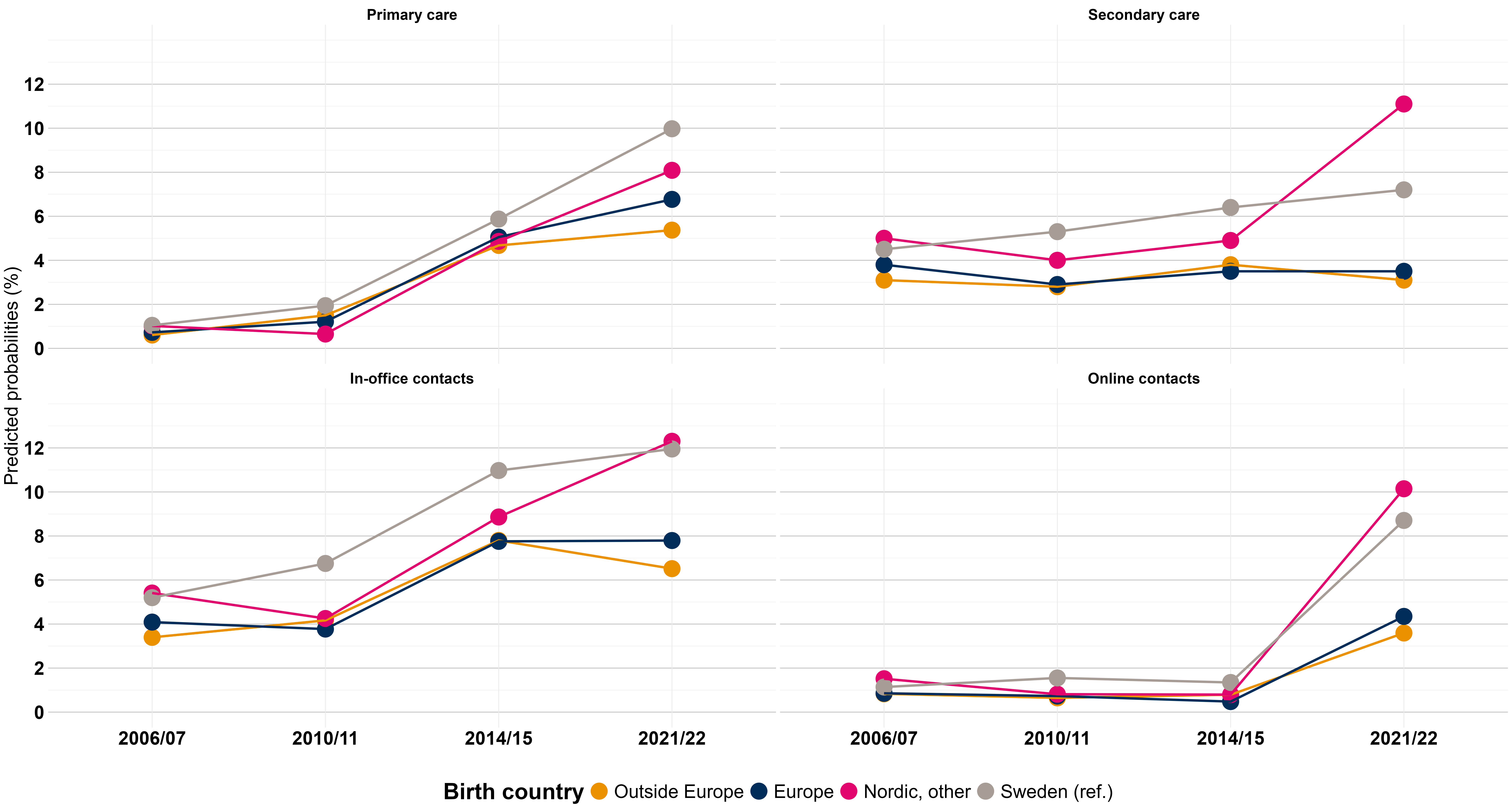
**

*Figure S1. Predicted probabilities of mental healthcare use by healthcare level and type of contact. Note: the sharp increase in mental healthcare use within primary healthcare is most likely due to improved primary care coverage over time.*

**
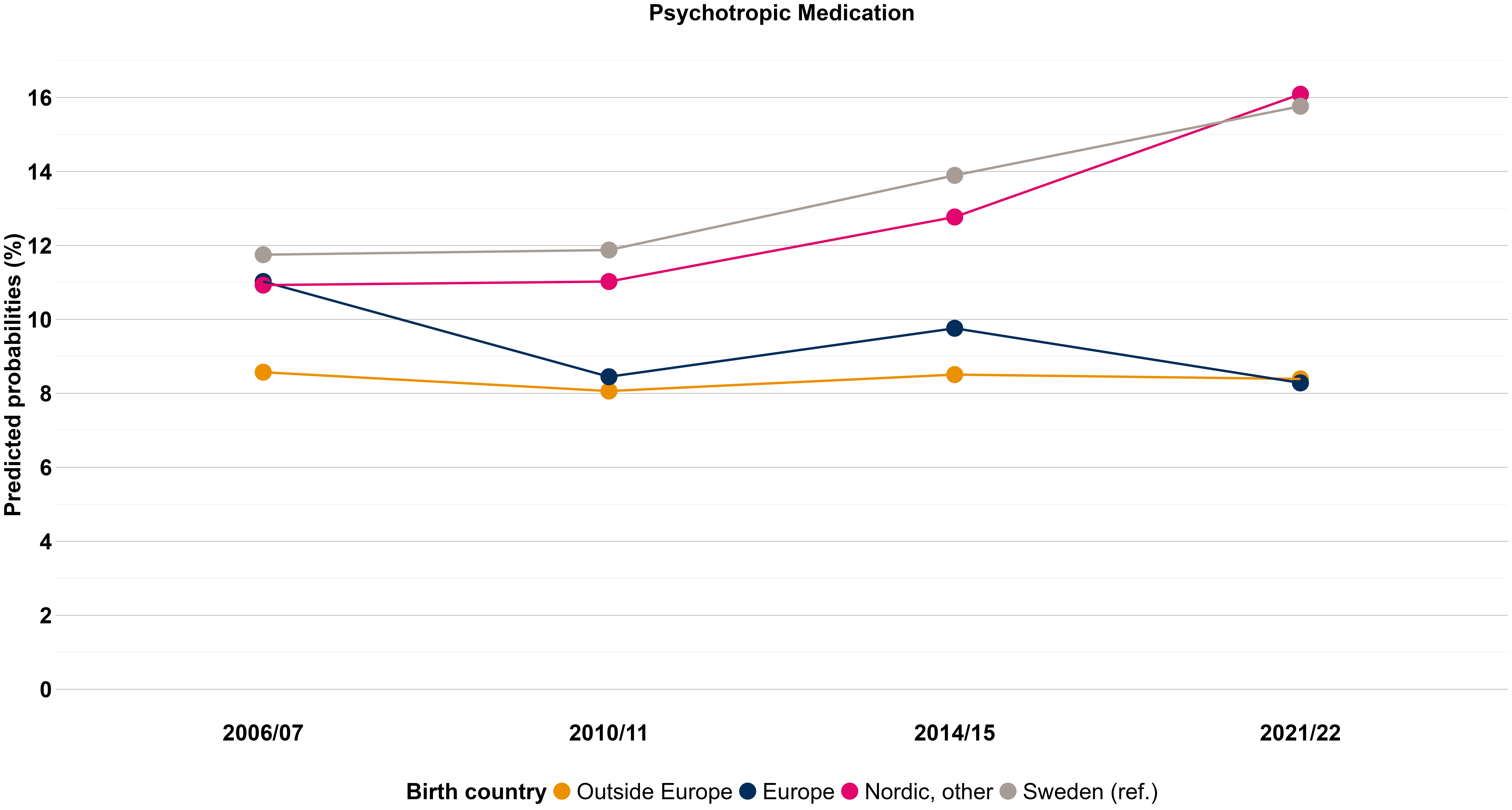
**

*Figure S2. Predicted probabilities of psychotropic medication collection across periods.*

**Table S1. Predicted probabilities of mental healthcare use by healthcare level and type of contact**

|  | **2006/2007** | **2010/2011** | **2014/2015** | **2021/2022** |
| --- | --- | --- | --- | --- |
| ***Primary care**** |  |  |  |  |
| Outside Europe | 0.6% (0.4%; 0.8%) | 1.5% (1.0%; 2.0%) | 4.7% (3.6%; 5.7%) | 5.4% (4.4%; 6.4%) |
| Europe | 0.7% (0.3%; 1.1%) | 1.2% (0.6%; 1.8%) | 5.1% (3.6%; 6.5%) | 6.8% (5.3%; 8.3%) |
| Nordic, other | 1.0% (0.5%; 1.6%) | 0.7% (0.2%; 1.1%) | 4.9% (2.8%; 6.9%) | 8.1% (4.4%; 11.8%) |
| Sweden | 1.0% (0.9%; 1.2%) | 1.9% (1.7%; 2.2%) | 5.9% (5.4%; 6.4%) | 10.0% (9.4%; 10.6%) |
| ***Specialized care*** |  |  |  |  |
| Outside Europe | 3.1% (2.4%; 3.8%) | 2.8% (2.1%; 3.5%) | 3.8% (2.9%; 4.7%) | 3.1% (2.3%; 3.8%) |
| Europe | 3.8% (2.7%; 4.9%) | 2.9% (2.0%; 3.8%) | 3.5% (2.3%; 4.8%) | 3.5% (2.3%; 4.6%) |
| Nordic, other | 5.0% (3.5%; 6.4%) | 4.0% (2.5%; 5.5%) | 4.9% (2.8%; 7.0%) | 11.1% (7.1%; 15.0%) |
| Sweden | 4.5% (4.1%; 4.8%) | 5.3% (4.9%; 5.7%) | 6.4% (5.8%; 6.9%) | 7.2% (6.6%; 7.7%) |
| ***Psychotropic medication*** |  |  |  |  |
| Outside Europe | 8.6% (7.4%; 9.7%) | 8.1% (6.9%; 9.2%) | 8.5% (7.1%; 9.9%) | 8.4% (7.1%; 9.6%) |
| Europe | 11.0% (9.3%; 12.7%) | 8.4% (6.9%; 10.0%) | 9.8% (7.8%; 11.7%) | 8.3% (6.6%; 10.0%) |
| Nordic, other | 10.9% (9.1%; 12.7%) | 11.0% (9.0%; 13.0%) | 12.8% (10.0%; 15.6%) | 16.1% (12.0%; 20.2%) |
| Sweden | 11.8% (11.3%; 12.2%) | 11.9% (11.3%; 12.4%) | 13.9% (13.2%; 14.6%) | 15.8% (15.0%; 16.5%) |
| ***Hospitalization*** |  |  |  |  |
| Outside Europe | 0.3% (0.1%; 0.5%) | 0.3% (0.0%; 0.5%) | 0.2% (0.0%; 0.4%) | 0.2% (0.0%; 0.4%) |
| Europe | 0.3% (0.0%; 0.6%) | 0.1% (0.0%; 0.1%) | 0.2% (-0.1%; 0.5%) | 0.3% (-0.1%; 0.7%) |
| Nordic, other | 0.2% (0.0%; 0.3%) | 0.5% (-0.1%; 1.2%) | - | 0.9% (-0.7%; 2.5%) |
| Sweden | 0.5% (0.4%; 0.6%) | 0.5% (0.4%; 0.6%) | 0.6% (0.4%; 0.8%) | 0.5% (0.4%; 0.7%) |
| ***Type of contact*** |  |  |  |  |
| ***Physical*** |  |  |  |  |
| Outside Europe | 3.4% (2.7%; 4.1%) | 4.2% (3.3%; 5.0%) | 7.8% (6.5%; 9.1%) | 6.5% (5.4%; 7.6%) |
| Europe | 4.1% (3.0%; 5.2%) | 3.8% (2.8%; 4.8%) | 7.8% (6.0%; 9.5%) | 7.8% (6.2%; 9.4%) |
| Nordic, other | 5.4% (3.9%; 6.9%) | 4.3% (2.8%; 5.7%) | 8.9% (6.1%; 11.6%) | 12.3% (8.1%; 16.5%) |
| Sweden | 5.2% (4.8%; 5.6%) | 6.8% (6.3%; 7.2%) | 11.0% (10.3%; 11.6%) | 12.0% (11.3%; 12.6%) |
| ***Digital#*** |  |  |  |  |
| Outside Europe | 0.8% (0.5%; 1.2%) | 0.6% (0.4%; 0.9%) | 0.8% (0.4%; 1.2%) | 3.6% (2.8%; 4.4%) |
| Europe | 0.9% (0.3%; 1.4%) | 0.7% (0.3%; 1.2%) | 0.5% (0.1%; 0.9%) | 4.3% (3.1%; 5.6%) |
| Nordic, other | 1.5% (0.7%; 2.3%) | 0.8% (0.2%; 1.4%) | 0.8% (-0.1%; 1.7%) | 10.1% (6.2%; 14.1%) |
| Sweden | 1.1% (1.0%; 1.3%) | 1.6% (1.3%; 1.8%) | 1.3% (1.1%; 1.6%) | 8.7% (8.1%; 9.3%) |
|  | - **- primary care record-coverage improved over time, therefore increases in probabilities are more likely due to improved record coverage.* - *# - a sharp increase in digital service utilization in 2021/2022 most likely related to system-level transition to online care during the Covid-19 Pandemic.* - *Estimates from logistic regression adjusted for adjusted for age, sex, income (quintiles), education (7 levels), and psychological distress (3 levels)* - *Weighted analysis* | | | |

## *Odds ratios comparing mental healthcare utilization between migrant groups and Swedish-born individuals (2006-2022)*

**Tabel S2. Odds ratios comparing mental healthcare use between migrant groups and Swedish-born individuals, 2006/2007 – 2021/2022**

|  | **2006/2007** | | | | **2010/2011** | | | | **2014/2015** | | | | **2021/2022** | | | |  |
| --- | --- | --- | --- | --- | --- | --- | --- | --- | --- | --- | --- | --- | --- | --- | --- | --- | --- |
|  | **Model1** | **Model2** | **Model3** | **Model4** | **Model1** | **Model2** | **Model3** | **Model4** | **Model1** | **Model2** | **Model3** | **Model4** | **Model1** | **Model2** | **Model3** | **Model4** | p-values, Wald |
|  | *ORs (95% CI)* | *ORs (95% CI)* | *ORs (95% CI)* | *ORs (95% CI)* | *ORs (95% CI)* | *ORs (95% CI)* | *ORs (95% CI)* | *ORs (95% CI)* | *ORs (95% CI)* | *ORs (95% CI)* | *ORs (95% CI)* | *ORs (95% CI)* | *ORs (95% CI)* | *ORs (95% CI)* | *ORs (95% CI)* | *ORs (95% CI)* |  |
| Outside Europe | 0.99 (0.86, 1.14) | 0.86 (0.75; 1.00)* | **0.72 (0.62; 0.85)** | **0.63 (0.53; 0.75)** | 0.94 (0.81, 1.09) | **0.70 (0.59; 0.81)** | **0.64 (0.54; 0.75)** | **0.57 (0.48; 0.69)** | 0.86 (0.74, 1.01)* | **0.59 (0.49; 0.70)** | **0.58 (0.49; 0.70)** | **0.57 (0.47; 0.70)** | **0.63 (0.55, 0.73)** | **0.53 (0.45; 0.62)** | **0.46 (0.39; 0.54)** | **0.49 (0.41; 0.58)** | <0.0001 |
| Europe | **1.23 (1.04, 1.45)** | 1.05 (0.88; 1.25) | 0.93 (0.77; 1.11) | **0.77 (0.63; 0.94)** | 0.89 (0.74, 1.07) | **0.71 (0.58; 0.85)** | **0.66 (0.54; 0.80)** | **0.58 (0.47; 0.71)** | 0.82 (0.67, 1.00)* | **0.63 (0.51; 0.79)** | **0.63 (0.50; 0.80)** | **0.68 (0.52; 0.87)** | **0.58 (0.48, 0.71)** | **0.54 (0.44; 0.66)** | **0.48 (0.39; 0.59)** | **0.47 (0.38; 0.59)** | <0.0001 |
| Nordic, other | **1.31 (1.09, 1.56)** | 0.89 (0.74; 1.07) | 0.91 (0.75; 1.10) | **0.79 (0.64; 0.98)** | **1.36 (1.12, 1.66)** | **0.70 (0.59; 0.81)** | 0.85 (0.68; 1.05)* | **0.74 (0.59; 0.93)** | 1.25 (0.97, 1.62)* | 0.92 (0.71; 1.19) | 0.93 (0.71; 1.22) | 0.97 (0.72; 1.30) | 1.19 (0.88, 1.61) | 1.04 (0.76; 1.43) | 1.07 (0.78; 1.48) | 0.97 (0.69; 1.37) | 0.9632 |
| Sweden (ref.) | 1 | 1 | 1 | 1 | 1 | 1 | 1 | 1 | 1 | 1 | 1 | 1 | 1 | 1 | 1 | 1 |  |
|  | - *Model1 – crude model* - *Model2 – adjusted for sex, age, income (quintiles), education (7 levels)* - ***Model3 (Main model) – adjusted for sex, age, income (quintiles), education (7 levels), and psychological distress (3 levels).*** *P-values shown* - *Model4 – adjusted for sex, age, income (quintiles), education (7 levels), psychological distress (3 levels), general health status (5 levels), and long-term limiting illness (binary).* - *Bold -statistically significant (p-value < 0.05); * borderline significant* | | | | | | | | | | | | | | | | |

## *Sex- and age-stratified analyses of differences in mental healthcare utilization between migrant groups and Swedish-born individuals (2006-2022)*

**Table S3. Odds ratio of mental healthcare use comparing migrant groups and Swedish-born individuals across surveys, stratified by sex**

|  | **2006/2007** | **2010/2011** | **2014/2015** | **2021/2022** |
| --- | --- | --- | --- | --- |
|  | *ORs (95% CI)* | *ORs (95% CI)* | *ORs (95% CI)* | *ORs (95% CI)* |
| **Sex** |  |  |  |  |
| ***Males*** |  |  |  |  |
| Outside Europe | **0.66 (0.51; 0.85)** | **0.78 (0.60; 1.01)** | **0.56 (0.42; 0.76)** | **0.46 (0.35; 0.61)** |
| Europe | 0.95 (0.71; 1.27) | **0.70 (0.51; 0.96)** | **0.57 (0.37; 0.86)** | **0.42 (0.28; 0.62)** |
| Nordic, other | 0.86 (0.59; 1.25) | 0.58 (0.36; 0.93) | 0.90 (0.54; 1.49) | 1.22 (0.68; 2.21) |
| Sweden (ref.) | 1 | 1 | 1 | 1 |
| ***Females*** |  |  |  |  |
| Outside Europe | **0.76 (0.63; 0.93)** | **0.56 (0.46; 0.69)** | **0.59 (0.47; 0.75)** | **0.46 (0.38; 0.57)** |
| Europe | 0.90 (0.71; 1.13) | **0.64 (0.50; 0.82)** | **0.67 (0.51; 0.88)** | **0.51 (0.40; 0.66)** |
| Nordic, other | 0.94 (0.75; 1.17) | 0.95 (0.74; 1.21) | 0.95 (0.69; 1.30) | 1.01 (0.69; 1.48) |
| Sweden (ref.) | 1 | 1 | 1 | 1 |
| **Age group** |  |  |  |  |
| ***Younger adults (18–29)*** |  |  |  |  |
| Outside Europe | 0.85 (0.55; 1.31) | **0.45 (0.27; 0.73)** | 0.59 (0.35; 1.00)* | **0.33 (0.20; 0.55)** |
| Europe | 1.08 (0.61; 1.91) | **0.50 (0.26; 0.98)** | 0.53 (0.25; 1.14) | **0.21 (0.10; 0.47)** |
| Nordic, other | 1.39 (0.62; 3.12) | 2.22 (0.80; 6.12) | 1.04 (0.25; 4.29) | 0.51 (0.15; 1.77) |
| Sweden (ref.) | 1 | 1 | 1 | 1 |
| ***Older adults (30–64)*** |  |  |  |  |
| Outside Europe | **0.66 (0.56; 0.77)** | **0.65 (0.55; 0.78)** | **0.56 (0.46; 0.68)** | **0.48 (0.40; 0.58)** |
| Europe | 0.93 (0.77; 1.13) | **0.70 (0.57; 0.87)** | **0.66 (0.52; 0.84)** | **0.52 (0.42; 0.65)** |
| Nordic, other | 1.03 (0.84; 1.25) | 1.04 (0.84; 1.28) | 1.08 (0.82; 1.42) | 1.22 (0.88; 1.70) |
| Sweden (ref.) | 1 | 1 | 1 | 1 |
|  | - *Adjusted for sex, age, income (quintiles), education (7 levels), and psychological distress (3 levels).* - *Bold -statistically significant (p-value < 0.05); * borderline significant* | | | |

## *Odds ratios comparing mental healthcare utilization between migrant groups and Swedish-born individuals by healthcare level and type of contact (2021/2022)*

**Table S4. Differences in mental healthcare use by healthcare level and type of contact in 2021/2022**

|  | **Primary care** | **Secondary outpatient care** | **Psychotropic** | **In-office visits** | **Online visits** |
| --- | --- | --- | --- | --- | --- |
|  | *ORs (95% CI)* | *ORs (95% CI)* | *ORs (95% CI)* | *ORs (95% CI)* | *ORs (95% CI)* |
| Outside Europe | **0.49 (0.39; 0.62)** | **0.38 (0.28; 0.51)** | **0.46 (0.38; 0.55)** | **0.48 (0.39; 0.60)** | **0.36 (0.28; 0.47)** |
| Europe | **0.64 (0.49; 0.83)** | **0.43 (0.30; 0.63)** | **0.45 (0.35; 0.58)** | **0.59 (0.46; 0.76)** | **0.45 (0.32; 0.62)** |
| Nordic, other | 0.78 (0.46; 1.33) | **1.71 (1.07; 2.71)** | 1.03 (0.73; 1.45) | 1.04 (0.67; 1.62) | 1.21 (0.74; 1.97) |
| Sweden (ref.) | 1 | 1 | 1 | 1 | 1 |
|  | - *Adjusted for sex, age, income (quintiles), education (7 levels), and psychological distress (3 levels).* - *Results are only for the 2021/2022 period because of too few cases of online mental healthcare use in previous periods.* - *Bold -statistically significant (p-value < 0.05); * borderline significant* - *Results for hospitilization/inpatient care not shown due to low statistical power due to very few cases.*   ***Post-hoc analysis:***  *Because access to secondary care is mainly referral-based, a post-hoc analysis examined migrant-related differences in secondary outpatient care among individuals in contact with primary care (note: this sample is small and has low statistical power; Outside Europe vs Sweden, OR = 0.45 [0.23-0.88], Europe vs Sweden, OR = 0.54 (0.26-1.10], and Nordic, other vs Sweden, OR = 1.35 [0.52-3.55]).* | | | | |

## *Predicted number of outpatient visits by group, conditional on at least one visit (2006-2022)*

**Table S5. Predicted number of outpatient visits (95% CI) by group over time, conditional on having at least one outpatient visit**

|  | **2006/2007** | **2010/2011** | **2014/2015** | **2021/2022** |
| --- | --- | --- | --- | --- |
| Outside Europe | 2.2 (1.3; 3.0) | 2.3 (1.4; 3.2) | 1.6 (0.8; 2.4) | 2.1 (1.3; 2.9) |
| Europe | 3.4 (1.7; 5.1) | 3.2 (1.4; 5.0) | 2.1 (0.9; 3.4) | 2.0 (1.2; 2.9) |
| Nordic | 3.4 (1.7; 5.1) | 2.1 (0.9; 3.3) | 1.1 (0.3; 1.9) | 3.2 (1.5; 4.9) |
| Sweden | 3.3 (2.4; 4.1) | 3.3 (2.4; 4.1) | 1.9 (1.1; 2.7) | 2.3 (1.7; 3.0) |
|  | *Adjusted for sex, age, income (quintiles), education (7 levels), and psychological distress (3 levels).* | | | |

## *Rate ratios comparing number of outpatient visits between migrant groups and Swedish-born individuals (2006-2022)*

**Table S6. Rate ratios comparing frequency of outpatient visits between migrant groups and Swedish-born individuals, conditional on having at least one outpatient visit (2006-2022)**

|  | **2006/2007** | | | | **2010/2011** | | | | **2014/2015** | | | | **2021/2022** | | | |  |
| --- | --- | --- | --- | --- | --- | --- | --- | --- | --- | --- | --- | --- | --- | --- | --- | --- | --- |
|  | **Model1** | **Model2** | **Model3** | **Model4** | **Model1** | **Model2** | **Model3** | **Model4** | **Model1** | **Model2** | **Model3** | **Model4** | **Model1** | **Model2** | **Model3** | **Model4** | p-values, Wald |
|  | *RR (95% CI)* | *RR (95% CI)* | *RR (95% CI)* | *RR (95% CI)* | *RR (95% CI)* | *RR (95% CI)* | *RR (95% CI)* | *RR (95% CI)* | *RR (95% CI)* | *RR (95% CI)* | *RR (95% CI)* | *RR (95% CI)* | *RR (95% CI)* | *RR (95% CI)* | *RR (95% CI)* | *RR (95% CI)* |  |
| Outside Europe | 0.75 (0.54, 1.03)* | 0.73 (0.52; 1.02)* | **0.67 (0.48; 0.93)** | **0.66 (0.47; 0.91)** | 0.75 (0.53, 1.08) | **0.70 (0.49; 0.99)** | 0.70 (0.48; 1.01)* | 0.76 (0.52; 1.09) | 0.82 (0.60, 1.11) | 0.83 (0.61; 1.13) | 0.85 (0.61; 1.17) | 0.77 (0.55; 1.07) | 0.87 (0.65, 1.17) | 0.93 (0.68; 1.27) | 0.90 (0.65; 1.26) | 0.96 (0.69; 1.34) | 0.7139 |
| Europe | 0.95 (0.59, 1.52) | 1.00 (0.64; 1.54) | 1.04 (0.67; 1.62) | 1.04 (0.66; 1.63) | 0.98 (0.57, 1.68) | 1.04 (0.61; 1.76) | 0.98 (0.57; 1.68) | 0.97 (0.54; 1.73) | 1.05 (0.69, 1.60) | 1.09 (0.69; 1.71) | 1.15 (0.75; 1.77) | 1.09 (0.69; 1.72) | 0.85 (0.60, 1.21) | 1.00 (0.70; 1.43) | 0.87 (0.64; 1.19) | 0.92 (0.68; 1.24) | 0.7829 |
| Nordic, other | 1.00 (0.60, 1.66) | 0.98 (0.64; 1.52) | 1.03 (0.66; 1.62) | 1.01 (0.66; 1.56) | **0.60 (0.37, 0.96)** | 0.67 (0.42; 1.07) | 0.65 (0.39; 1.06) | 0.71 (0.43; 1.18) | 0.55 (0.29, 1.05)* | **0.53 (0.28; 0.99)** | 0.57 (0.30; 1.09) | **0.52 (0.29; 0.95)** | 1.64 (0.82, 3.30) | 1.46 (0.89; 2.38) | 1.37 (0.87; 2.17) | 1.32 (0.83; 2.11) | 0.0946 |
| Sweden (ref.) | 1 | 1 | 1 | 1 | 1 | 1 | 1 | 1 | 1 | 1 | 1 | 1 | 1 | 1 | 1 | 1 |  |
|  | - *Model1 – crude model* - *Model2 – adjusted for sex, age, income (quintiles), education (7 levels)* - ***Model3 (Main model) – adjusted for sex, age, income (quintiles), education (7 levels), and psychological distress (3 levels).*** *P-values shown* - *Model4 – adjusted for sex, age, income (quintiles), education (7 levels), psychological distress (3 levels), general health status (5 levels), and long-term limiting illness (binary).* - *RR – Rate ratio* - *Bold -statistically significant (p-value < 0.05); * borderline significant* | | | | | | | | | | | | | | | | |

## *Sensitivity analysis comparing results based on complete case analysis and methods for handling missing data*

**Table S7. Sensitivity analysis using item missing as categories and using multiple imputations (performed due to 9.3% item missing on the general health status in 2014/2015)**

|  | **2006/2007** | | | **2010/2011** | | | **2014/2015** | | | **2021/2022** | | |
| --- | --- | --- | --- | --- | --- | --- | --- | --- | --- | --- | --- | --- |
|  | **CCA** | **Missing as category** | **Imputed** | **CCA** | **Missing as category** | **Imputed** | **CCA** | **Missing as category** | **Imputed** | **CCA** | **Missing as category** | **Imputed** |
|  | *ORs (95% CI)* | *ORs (95% CI)* | *ORs (95% CI)* | *ORs (95% CI)* | *ORs (95% CI)* | *ORs (95% CI)* | *ORs (95% CI)* | *ORs (95% CI)* | *ORs (95% CI)* | *ORs (95% CI)* | *ORs (95% CI)* | *ORs (95% CI)* |
| Outside Europe | **0.65 (0.54; 0.77)** | **0.62 (0.52; 0.73)** | **0.61 (0.52; 0.72)** | **0.59 (0.49; 0.70)** | **0.61 (0.51; 0.72)** | **0.59 (0.49; 0.70)** | **0.58 (0.48; 0.71)** | **0.54 (0.45; 0.66)** | **0.54 (0.45; 0.65)** | **0.48 (0.41; 0.57)** | **0.48 (0.41; 0.57)** | **0.48 (0.41; 0.57)** |
| Europe | **0.78 (0.64; 0.96)** | **0.77 (0.63; 0.94)** | **0.76 (0.63; 0.92)** | **0.59 (0.48; 0.73)** | **0.58 (0.47; 0.72)** | **0.58 (0.47; 0.72)** | **0.68 (0.53; 0.87)** | **0.59 (0.47; 0.76)** | **0.60 (0.47; 0.76)** | **0.48 (0.39; 0.60)** | **0.48 (0.39; 0.60)** | **0.49 (0.39; 0.60)** |
| Nordic, other | **0.78 (0.63; 0.96)** | **0.78 (0.63; 0.96)** | **0.77 (0.62; 0.95)** | **0.76 (0.60; 0.95)** | **0.79 (0.63; 0.99)** | **0.75 (0.60; 0.93)** | 0.97 (0.72; 1.30) | 0.89 (0.67; 1.18) | 0.93 (0.70; 1.23) | 0.95 (0.68; 1.33) | 0.97 (0.70; 1.35) | 0.95 (0.69; 1.32) |
| Sweden (ref.) | 1 | 1 | 1 | 1 | 1 | 1 | 1 | 1 | 1 | 1 | 1 | 1 |
|  | - *CCA – Complete Case Analysis* - *Model 4 results adjusting for sociodemographic factors and all need indicators.* - *The total sample is 81650 over the four periods: 27 754 in 2006; 22 463 in 2010; 15 490 in 2014; and 15 943 in 2021.* - *Variables with missing that were imputed include: income (11 missing of 81 650, only in 2021), education (458 missing), distress (840 missing), general health status (1992 missing; mostly in 2014), long-term limiting illness (724 missing), and migrant status (9 missing of 81650)* - *Bold -statistically significant (p-value < 0.05); * borderline significant* | | | | | | | | | | | |

## *Mean of psychological distress among mental healthcare users by migrant status (2006-2022)*

To explore differences in likely mental healthcare needs among service users, we examined distress levels across groups and found that migrant groups consistently reported higher distress than Swedish-born individuals. This suggests that non-Nordic migrants contact care much later than Swedish-born individuals.

**Table S8. The mean of psychological distress among service users by migrant status and period**

|  | **2006/2007 (GHQ-12)** | **2010/2011 (GHQ-12)** | **2014/2015 (GHQ-12)** | **2021/2022 (Kessler 6)** |
| --- | --- | --- | --- | --- |
|  | *Mean(sd)* | *Mean(sd)* | *Mean(sd)* | *Mean(sd)* |
| Outside Europe | 4.24 (4.00) | 3.80 (4.20) | 4.07 (4.18) | 9.86 (6.35) |
| Europe | 3.87 (3.90) | 3.99 (4.39) | 3.91 (4.16) | 8.98 (6.35) |
| Nordic, other | 2.60 (3.45) | 2.86 (4.09) | 3.33 (4.13) | 7.50 (5.26) |
| Sweden (ref.) | 2.74 (3.59) | 2.80 (3.68) | 3.53 (3.93) | 7.76 (5.31) |

## *Robustness checks due to change of instrument from GHQ-12 to Kessler 6*

As discussed in the manuscript, Kessler 6 is a better proxy of mental healthcare needs than GHQ-12. In this study Kessler 6 was used in 2021/2022 study period which means that the instrument change does affect the equity estimates (need adjusted inequalities). The following checks were done to test the robustness of the changes in migrant-inequality between 2014/2015 and 2021/2022. All models, A-F, are comparing the likelihood of mental healthcare use between migrant groups and Swedish-born individuals using odds ratios.

**Table S9 A-F**

1. **Models adjusted for sociodemographic factors and long-term limiting illness and self-rated general health status, mesured consistently over the study periods**

|  | **2014/2015** | **2021/2022** |
| --- | --- | --- |
|  | *ORs (95% CI)* | *ORs (95% CI)* |
| **Outside Europe** | **0.55 (0.44, 0.67)** | **0.52 (0.44, 0.62)** |
| **Europe** | **0.65 (0.51, 0.84)** | **0.51 (0.41, 0.64)** |
| **Nordic, other** | 0.98 (0.73, 1.31) | 0.92 (0.66, 1.28) |
| **Sweden (ref.)** |  |  |

1. **Models adjusted for sociodemographic factors and raw distress scores (continuous) without equating using crosswalks**

|  | **2014/2015 (GHQ-12)** | **2021/2022 (Kessler 6)** |
| --- | --- | --- |
|  | *ORs (95% CI)* | *ORs (95% CI)* |
| **Outside Europe** | **0.58 (0.48, 0.70)** | **0.43 (0.36, 0.52)** |
| **Europe** | **0.65 (0.51, 0.81)** | **0.47 (0.38, 0.58)** |
| **Nordic, other** | 0.93 (0.71, 1.22) | 1.05 (0.75, 1.47) |
| **Sweden (ref.)** |  |  |

1. **Models adjusted for sociodemographic factors and binary cut-offs based on crosswalks (≥3 on the GHQ-12/≥8 on the Kessler 6)**

|  | **2014/2015 (GHQ-12)** | **2021/2022 (Kessler 6)** |
| --- | --- | --- |
|  | *ORs (95% CI)* | *ORs (95% CI)* |
| **Outside Europe** | **0.59 (0.49, 0.71)** | **0.47 (0.40, 0.55)** |
| **Europe** | **0.66 (0.52, 0.82)** | **0.48 (0.39, 0.60)** |
| **Nordic, other** | 0.95 (0.73, 1.23) | 1.04 (0.75, 1.46) |
| **Sweden (ref.)** |  |  |

1. **Model adjusted for sociodemographic factors and binary cut-off based on crosswalks (severe distress: ≥8 on the GHQ-12 or ≥13 on the Kessler 6)**

|  | **2014/2015 (GHQ-12)** | **2014/2015 (GHQ-12)** |
| --- | --- | --- |
|  | *ORs (95% CI)* | *ORs (95% CI)* |
| **Outside Europe** | **0.59 (0.49, 0.71)** | **0.48 (0.41, 0.57)** |
| **Europe** | **0.64 (0.51, 0.80)** | **0.49 (0.40, 0.60)** |
| **Nordic, other** | 0.92 (0.70, 1.20) | 1.07 (0.78, 1.48) |
| **Sweden (ref.)** |  |  |

1. **Models using distress deciles (10 groups of equal sizes) instead of values, small random number added to remove ties before creating deciles**

|  | **2014/2015 (GHQ-12)** | **2014/2015 (GHQ-12)** |
| --- | --- | --- |
|  | *ORs (95% CI)* | *ORs (95% CI)* |
| **Outside Europe** | **0.58 (0.49, 0.70)** | **0.47 (0.40, 0.55)** |
| **Europe** | **0.64 (0.51, 0.81)** | **0.49 (0.40, 0.61)** |
| **Nordic, other** | 0.95 (0.73, 1.24) | 1.08 (0.77, 1.51) |
| **Sweden (ref.)** |  |  |

1. **Models using the distress rank (individuals ranked from lowest distress to highest distress), small random number added to remove ties before ranking**

|  | **2014/2015** | **2021/2022** |
| --- | --- | --- |
|  | *ORs (95% CI)* | *ORs (95% CI)* |
| **Outside Europe** | **0.58 (0.49, 0.70)** | **0.47 (0.40, 0.55)** |
| **Europe** | **0.64 (0.51, 0.81)** | **0.49 (0.40, 0.60)** |
| **Nordic, other** | 0.95 (0.73, 1.24) | 1.07 (0.77, 1.50) |
| **Sweden (ref.)** |  |  |
